# Remobilization of polychlorinated biphenyls from sediment and its consequences for their transport in river waters

**DOI:** 10.1007/s10661-012-2882-8

**Published:** 2012-09-25

**Authors:** Monika Gdaniec-Pietryka, Agata Mechlińska, Lidia Wolska, Agnieszka Gałuszka, Jacek Namieśnik

**Affiliations:** 1 Department of Analytical Chemistry, Chemical Faculty, Gdańsk University of Technology (GUT), 11/12G. Narutowicz St., 80-233 Gdańsk, Poland; 2 Interdepartmental Institute of Maritime and Tropical Medicine, Department of Environmental Toxicology, Medical University of Gdańsk, 9b Powstania Styczniowego St., 81-519 Gdynia, Poland; 3 Geochemistry and the Environment Div., Institute of Chemistry, Jan Kochanowski University, 15G Świętokrzyska St., 25-406 Kielce, Poland

**Keywords:** Polychlorinated biphenyls, Sediments, Water, Desorption, GC–MS, Sediment/water partition coefficient

## Abstract

A laboratory experiment was performed to examine the remobilization of indicator polychlorinated biphenyls (iPCBs) from sediments and its results were applied to the real-world data for explaining the transport of PCBs in river. Seven PCB concentrations were determined in three series of model water–sediment systems (3 g of river sediment, three different volumes of distilled water (0.5, 0.25, and 0.15 ml), and 5 mg of biocide) after 11 days of incubation. Solid-phase extraction was used for separation of analytes from the aqueous phase and solvent extraction for isolation of analytes from the sediments, respectively. The extracts were analyzed for individual iPCB congeners using gas chromatography–mass spectrometry method. For each series of the experiment, the concentrations of PCBs in aqueous phase were similar. The average sediment/water partition coefficient value was 10^4^ l/kg. The solubility of individual PCB congeners in water did not influence the desorption of PCBs from the sediment. Although the dominant form of PCBs in a water–sediment system occurs as suspended and colloidal fractions, these compounds are transported mostly in a dissolved form. Suspended and colloidal matter is a major sink for PCBs in low-energy aquatic environments. In contrast, the dissolved PCBs are readily transported in running waters. The mobilization of PCBs from sediments to aqueous phase, with respect to their solubility in water, seems to be limited, thus reducing the risk of secondary pollution.

## Introduction

The polychlorinated biphenyls (PCBs) belong to the most persistent, bioaccumulative, and toxic pollutants (Gdaniec-Pietryka et al. 2009; Eljarrat and Barceló 2009). These compounds were first synthesized in 1881 by Schmidt and Schultz (Cairns and Siegmund 1981) and since the early 1930s, their mixtures have found various commercial applications (capacitors, transformers, heat transfer systems, electrical insulating and hydraulic fluids, paints, coatings, flame retardants, etc; Erickson and Kaley 2011). Of the 209 possible PCB congeners, seven compounds (#28, 52, 101, 118, 153, 138, and 180) occur in highest concentrations in both biotic and abiotic matrices, and are routinely determined in environmental laboratories around the world (Babut et al. 2009). These compounds are often referred to as “indicator” PCBs and their total concentrations are indicative of contamination with PCBs (Storelli and Perrone 2010).

Despite the fact that many studies have been carried out to assess the PCB concentrations in freshwater and marine sediments (e.g., Khim et al. 1999; Müller et al. 1999; Eljarrat et al. 2001), there are still relevant gaps in the knowledge about the mobility and the complex geochemical behavior of PCBs in the water–sediment system. When hydrophobic compounds like PCBs are discharged into the aquatic environment, they may be either adsorbed on suspended particles in the water column or deposited on the sediment surface (Mechlińska et al. 2010). Subsequently, they may remain adsorbed, be converted into a dissolved form, or be bioaccumulated in aquatic organisms and possibly biomagnified in the food chain (Schuler and Lydy 2001; Leppänen et al. 2003; Cornelissen et al. 2005; Nasr et al. 2010). The PCB compounds that occur in the lower sediment layer are not transported further. Adsorbed onto the surface of the active sediment layer, these compounds are more prone to degradation becoming more soluble in water after microbiological degradation. This process leads to reduction in their molecular mass (McDonough and Dzombak 2005). An understanding of these processes is of crucial significance in an assessment of the risk posed to the aquatic environment by hydrophobic pollutants.

Sorption is a key process that determines the environmental fate of hydrophobic organic pollutants in surface waters (Mechlińska et al. 2009). It is commonly believed that xenobiotics adsorbed on sediment grains or suspended matter and colloids are not directly accessible to living organisms (and therefore have no toxic effect), do not undergo photochemical or microbiological degradation, and do not evaporate. Their transport through different environmental compartments is thus limited as opposed to dissolved forms. Sorption should therefore be considered as a process governing the environmental fate of these compounds (Amymarie and Gschwend 2002; Jonker 2004).

Sediments are a main sink for hydrophobic and persistent organic pollutants. However, following reduced emissions of PCBs, historically polluted sediments are now recognized as significant secondary pollution sources in many ecosystems, and a better understanding of the pollutant transport from the sediment to the water column is important in making environmental risk assessments and decisions about remedial actions (Hedman et al. 2009). Due to their considerable affinity for sediment grains and their resistance to chemical and biochemical degradation, PCBs have accumulated in aquatic sediments in relatively large quantities. The PCB concentrations of several micrograms per kilogram have been measured in sediment samples from various parts of the world (Kocan et al. 2001; Tam and Yao 2002; Tashiro et al. 2004).

The question of when adsorbed compounds undergo desorption, i.e., when they are remobilized, is a critical issue that needs constant monitoring. In contrast to the mechanism of sorption, the process of desorption is much less known (Gong et al. 1998; Gong and Depinto 1998). Remobilization of pollutants is expected within a limited range under certain conditions, both natural (e.g., tidal movement and storms) and anthropogenic (dredging, dredge disposal, and fishing; Eggleton and Thomas 2004). Desorption is thus possible, leading to secondary pollution of the environment by these compounds (Cornelissen et al. 1997; Björklund et al. 2000). In this study, we present the results of a laboratory experiment model of PCB desorption from sediment samples during their incubation. In addition, we use the data derived from the PCB determinations of the Odra River water samples collected during the International Odra Project (IOP) to explain the PCBs mobility in the river waters.

## Materials and methods

### Reagents and standards

The solvents used during the study were dichloromethane (99.9 %), methanol (99.8 %) and acetone (99.9 %) from Merck (Germany). Copper powder and silica gel were supplied by J.T. Baker. Individual solutions of seven PCB congeners (IUPAC #28, 52, 101, 118, 153, 138, and 180) were obtained from Restek Corporation (Bellefonte, USA) as 10 μg/ml solutions in isooctane. The internal standard, PCB 209 in isooctane (200 μg/ml), was purchased from Dr Ehrenstorfer GmbH (Germany). The stock solution of PCBs was prepared by mixing solutions (100 μl each) of these compounds. METRANAL^TM^2 and natural samples (surface sediments and water samples collected alongside the Odra River) were used as a certified reference material. The river sediment standard reference material (METRANAL^TM^2) was purchased from Analytica (Czech Republic) with certified concentrations of seven PCB congeners presented in Table 1.

**Table 1:** Certified analyte contents in the standard reference material METRANAL TM2

Analyte	Concentration (μg/kg)^a^	Uncertainty (μg/kg)^b^
Anthracene	393	128
Benzo(*a*)anthracene	998	260
Benzo(*b*)fluoranthene	829	228
Benzo(*k*)fluoranthene	423	116
Benzo(*g*,*h*,*i*)perylene	546	156
Benzo(*a*)pyrene	742	222
Dibenzo(*a*,*h*)anthracene	89	34
Fluoranthene	1,995	572
Chrysene	831	234
Indeno (1,2,3-cd)pyrene	585	166
Phenathrene	982	274
Pyrene	1677	424
PCB 28	23.3	6.8
PCB 31	16.3	5.0
PCB 52	29.2	9.0
PCB 77	–	–
PCB 101	28.1	7.6
PCB 110	17.4	5.4
PCB 118	12.2	3.2
PCB 138	61.3	15.4
PCB 149	50.6	12.6
PCB 153	70.2	19.8
PCB 163	20.1	6.2
PCB 170	33.4	8.8
PCB 180	63.6	15.4
PCB 187	30.8	7.8
PCB 194	14.2	4.0
Pentachlorobenzene	–	–
Hexachlorobenzene	45.1	11.2
2,4′-DDE	–	–
4,4′-DDE	19.0	5.6
4,4′-DDD	28.9	7.0
4,4′-DDT	45.8	12.4

^a^Dry mass at 40 °C

^b^Expanded combined uncertainty (*K* = 2)

### Analysis of extract samples

All experiments were performed using a TraceGC gas chromatograph (ThermoQuest, Finningan) equipped with a TraceMS mass spectrometric detector (ThermoQuest, Finningan) and an on-column injector. The capillary column used was a Rtx-5MS (30 m; 0.25 mm i.d. and 0.25 μm film thickness, 5 % phenyl, 95 % dimethylpolysiloxane). The carrier gas (helium) was maintained at constant pressure of 70 kPa. The gas chromatography (GC) oven temperature was programmed as follows: from 40 to 120 °C at a rate of 40 °C min^−1^, and to 280 °C at a rate of 5 °C min^−1^ where it was held for 12 min. The mass spectrometry (MS) operated in electron ionization mode with ion source temperature of 220 °C. The mass spectrometer operated on a selected ion monitoring mode; the following ions were monitored: (*m*/*z*) 256, 258, 290, 292, 326, 328, 360, 362, 394, 396, 494, and 496. An injection volume of 2 μl was selected for all analyses. The interface temperature was held at 280 °C.

### Desorption of PCB analytes from sediments

**Fig. 1:**
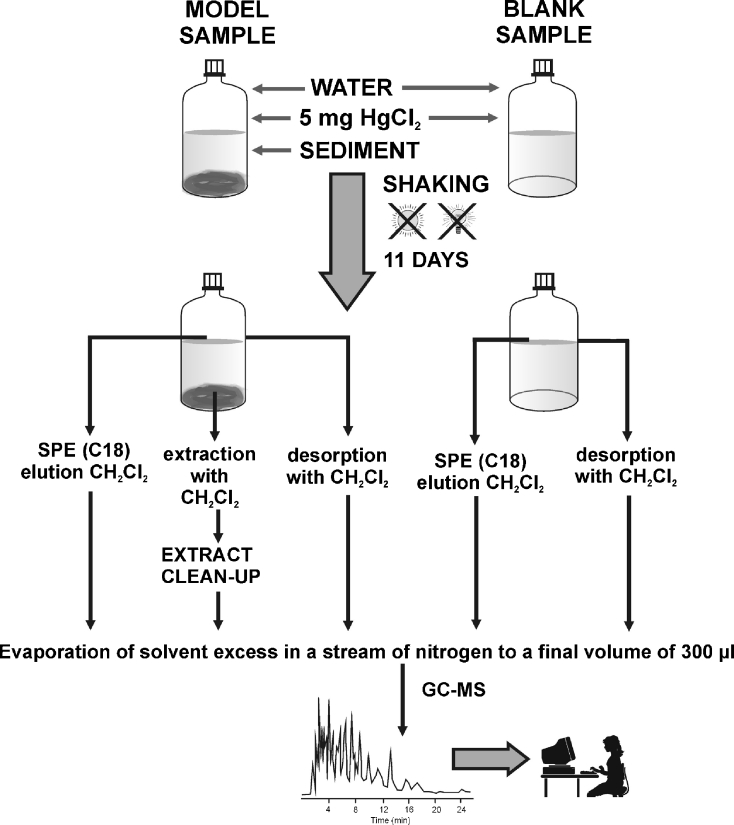
Schematic diagram of the procedure for testing PCB desorption from the model and blank samples

**Fig. 2:**
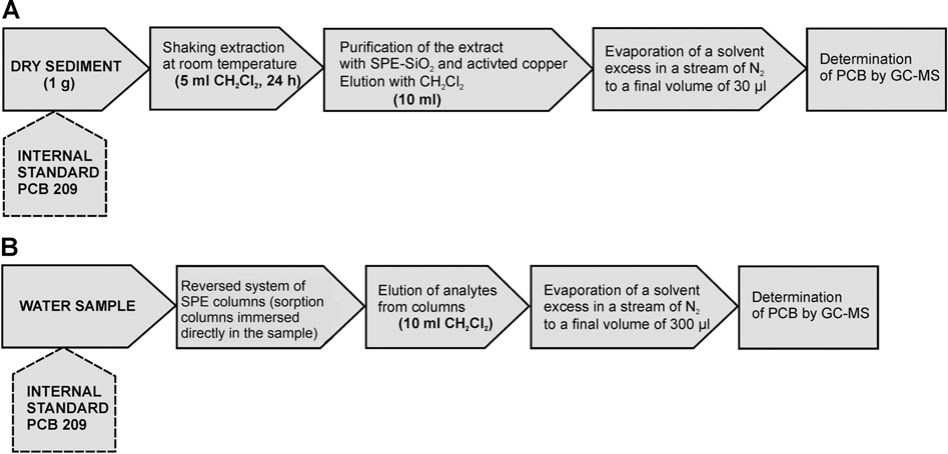
Schematic diagram of the procedure used during the testing of PCB desorption; **a** from the sediment samples, **b** from the water samples

Model studies were conducted in order to check the efficiency of PCB desorption from sediments. The experiments involved the application of model water–sediment systems stored in glass bottles. Three series of model systems were prepared in 1-l glass bottles with dry sediment samples (3 g) and distilled water in different proportions (0.50, 0.25, and 0.15 l) together with about 5 mg HgCl_2_ as a biocide. Five samples and one blank (without sediment) were prepared in each series. The samples were stored in dark places at a constant temperature for 11 days and rotated manually every day. The diagram of the procedure for testing PCB desorption from sediment samples is shown in Fig. 1. After 11 days of incubation, the analytes were extracted from the aqueous phase and the sediment. Analytes retained in the aqueous phase were separated using solid-phase extraction (SPE), while those present in the sediment were isolated by solvent extraction. The diagram of this procedure is presented in Fig. 2. To avoid the loss of analyte due to its adsorption to the walls of the bottles that enabled the water samples to be transported to the SPE columns, a reversed system was applied in which the sorption columns were directly immersed in the sample. In addition, the walls of the dry, empty bottles were rinsed with dichloromethane (20 ml) and the extract was collected in a vial. Subsequently, the solvent excess was evaporated in a stream of neutral gas (nitrogen):

1. aqueous phase extract and bottle wall rinsing extract–to a final volume of 300 μl,
2. the sediment extract–to a final volume of 1 ml.

The extract samples were analyzed by GC–MS (for operating parameters, see “Analysis of extract samples” section) in order to estimate the quantities of individual analytes.

The partition coefficients in the model sediment/water system for PCBs were calculated on the basis of analyte concentrations in various phases of the model system, employing the following formula:

$$ {K_D} = \frac{{{C_S}}}{{{C_W}}} $$

where, *K*_*D*_ − analyte partition coefficient,

*C*_*S*_: concentration of analyte remaining in the sediment after desorption
*C*_*W*_: concentration of analyte desorbed from the sediment in the extract

### Preparation of natural water and sediment samples for analysis

**Fig. 3:**
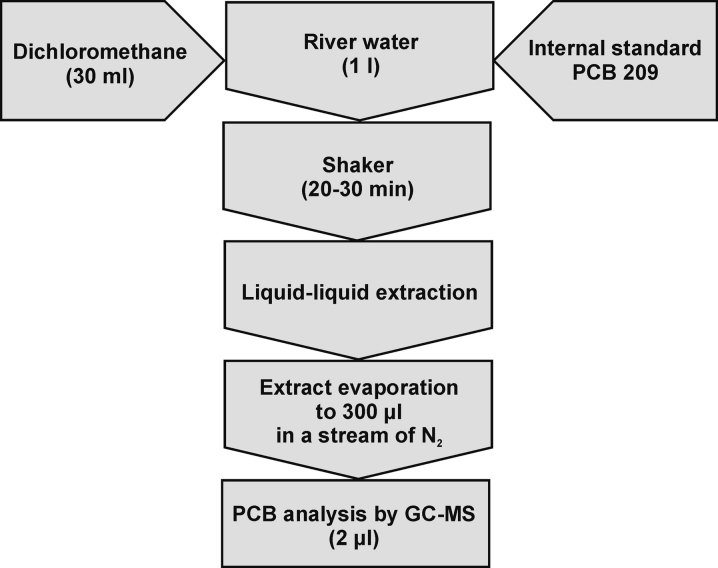
Schematic diagram of the procedure for determining PCB analytes in water samples

**Fig. 4:**
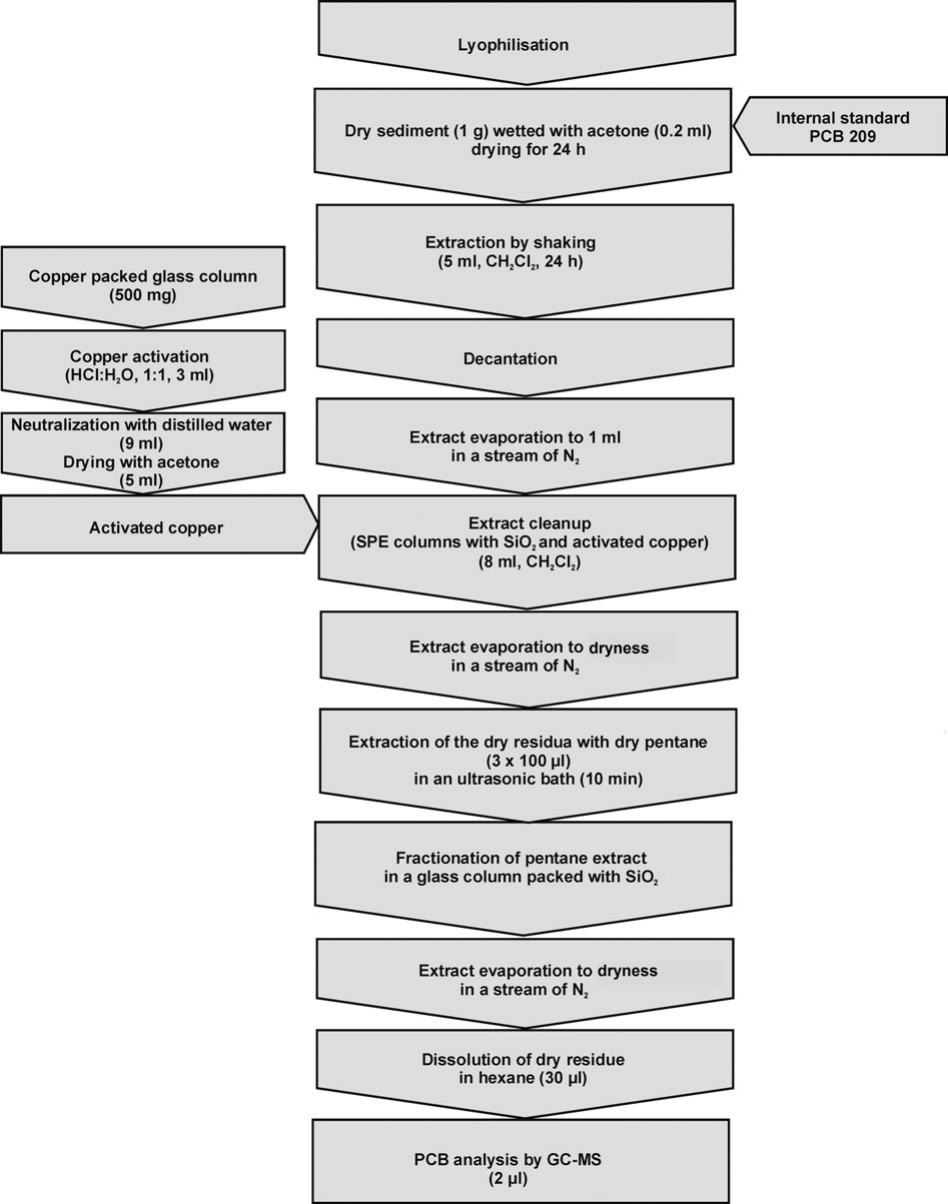
Schematic diagram of the procedure for determining PCB analytes in sediment samples

The analytical procedures used for a determination of PCB analytes in water and sediment samples are presented in Figs. 3 and 4. The physical speciation analysis was conducted on several bottom sediment and surface water samples collected from the Odra River. The sediment samples were collected with a Van Veen’s grab whereas the water samples were collected at a depth of ∼1 m above the bottom bed with a bathometer. The sediment samples were immediately frozen at temperature of about −20 °C.

The study within the framework of the IOP started in 1997 and lasted until 2000. The main objective of the IOP was to determine the concentrations, trend dynamics, and metabolic pathways of many types of pollutants in the Odra River basin. The study was conducted under normal and flood conditions.

## Results and discussion

### Model study of the desorption of PCB analytes from sediment samples

The partition coefficients of PCB analytes for the model water–sediment interaction system were calculated on the basis of the PCB contents determined in the aqueous phase (including analytes adsorbed on the bottle walls) and the sediment. These values were calculated using two possible approaches:

1. on the basis of analyte concentrations determined in each model system phase (concentration coefficient)
2. on the basis of absolute analyte concentrations determined in individual phases of the system (dimensionless partition coefficient for the whole system)

**Fig. 5:**
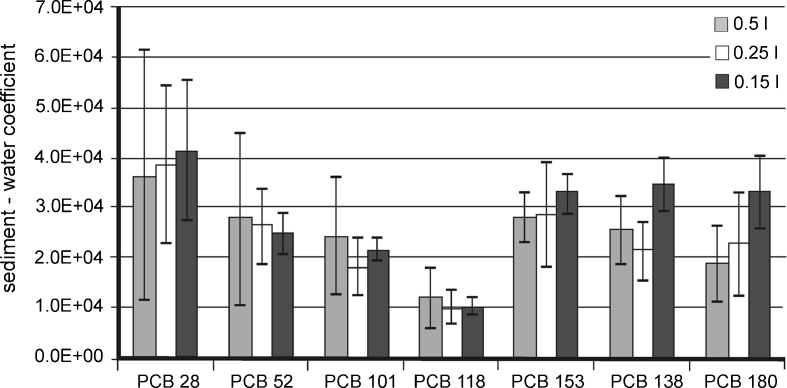
Comparison of sediment/water partition coefficients calculated on the basis of PCB contents determined in extracts obtained during the desorption of analytes from sediment samples

**Fig. 6:**
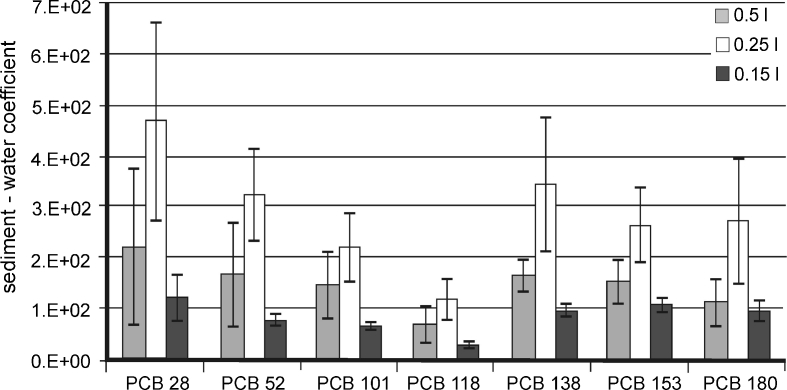
Comparison of dimensionless sediment/water partition coefficients calculated on the basis of PCB contents determined in extracts obtained during the desorption of analytes from sediment samples

The partition coefficients calculated for PCB analytes are presented in Figs. 5 and 6. These values are similar to those obtained by other authors and are not significantly higher than those estimated for the River Odra waters (Ghosh et al. 2000; Wolska et al. 1999).

The calculated values of sediment/water partition coefficients for PCBs averaged 10^4^ l/kg. They are comparable to values obtained by other authors (Galer et al. 2000; Witt 1995; Pörschmann et al. 1998). These results may suggest that the isolation technique (water decanted directly from the sediment into the SPE column) cannot ensure that the dissolved form of the target compound is separated from the form associated with the suspended and colloidal fraction. Despite every precaution that was taken during isolation, a small amount of suspended matter and colloids may get into the column with the water sample. This may, in turn, increase the concentration of target analytes in the aqueous phase (dissolved form), thereby reducing the partition coefficient.

**Fig. 7:**
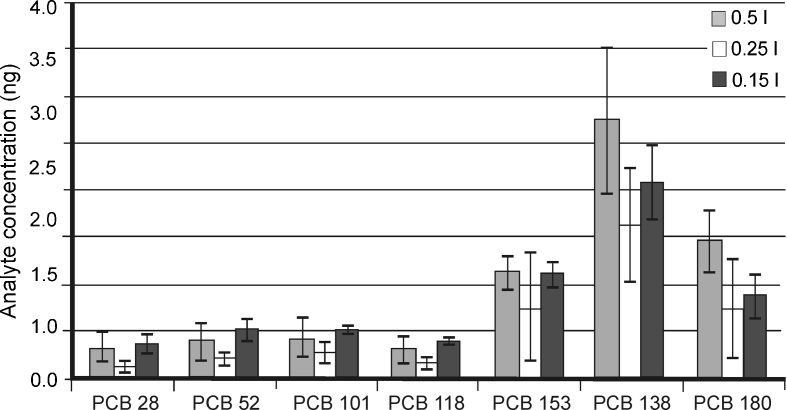
The amounts of PCBs desorbed from sediment samples during the model experiment carried out in the sediment–water system

Figure 7 provides information regarding the amounts of PCB analytes desorbed from the sediments during the experiment (for three series differing in their water content—0.15, 0.25, 0.5 l in the model system). The contents of the analytes determined in the aqueous phase were very similar in every case. This supports the hypothesis that the concentration of PCBs in the aqueous phase is, in fact, the sum of the concentration of the dissolved form and that associated with suspended and colloidal matter.

**Fig. 8:**
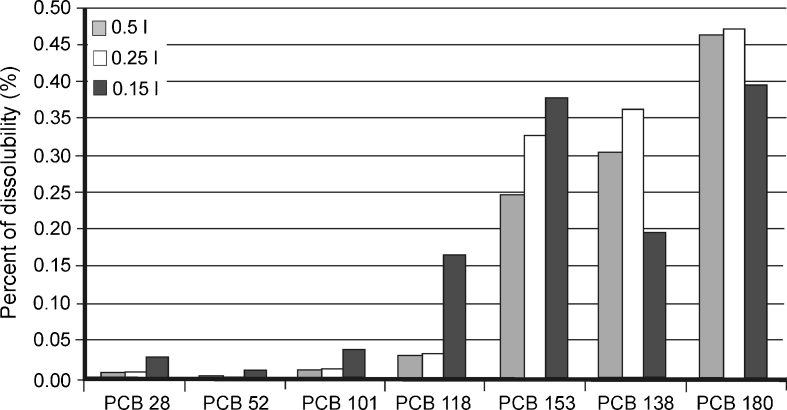
Concentrations of PCBs determined in water (after desorption from sediment) in comparison with their solubility (in percent)

In order to check whether the water solubility of PCB analytes increased their desorption efficiency, the quantity of analytes desorbed in a system was compared with the theoretical amount that could dissolve in a given volume of water at 20 °C. The results of these calculations, expressed as the percentage of the solubility of an analyte in water, are presented in Fig. 8.

Based on these results, it may be assumed that the solubility in water is not a factor that limits the desorption of PCB analytes from the sediment samples in this system. The analyte concentrations determined in water (after desorption from sediment) were lower than 0.5 % of the theoretical amount of analyte that can be dissolved in a given water volume. These amounts seem to be overestimated due to the fact that the isolation technique (SPE) used in this experiment does not ensure the isolation of dissolved forms of these compounds from water samples.

### Transport of PCB analytes in the aquatic environment—study of natural samples

**Fig. 9:**
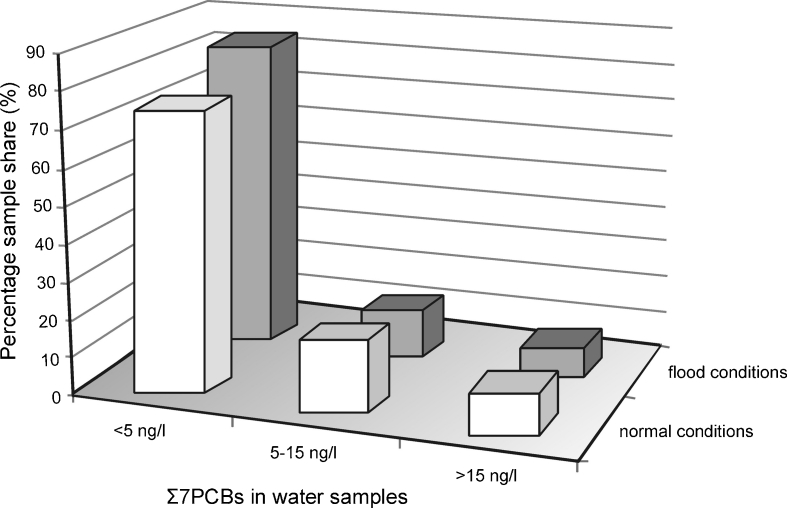
Percentage of samples characterized by a given level of the sum of seven PCBs in river waters during the flood and normal water level

The sediment/water partition coefficient of PCBs is high, but this study showed that it is roughly 1 order of magnitude lower than that of other lipophilic compounds, such as polycyclic aromatic hydrocarbons (PAHs; Helios-Rybicka et al. 2002; Wolska and Namieśnik 2002a, b; Jiang et al. 2000). This evidence suggests that PCBs are transported in the river water not only by suspended and colloidal fractions, but also by an aqueous phase (dissolved form). Müller (1998) reported a substantially higher amount of suspended matter in the Odra River during the flood in 1997 in comparison with normal conditions. He also observed higher PAH (650 kg) and lower PCB (1.6 kg) loads during flood than monthly loads of these compounds in 1996 (4.1 kg of PCBs and 426 kg of PAHs). The comparison of the overall percentage of seven PCBs determined in the Odra River water samples collected during flooding (August and November 1997) with that of similar samples collected in subsequent campaigns (May and November 1998, June 1999, May 2000) did not reveal any relationship between PCB concentrations and the amount of suspended matter (water samples collected during the flood contained significantly higher amounts of suspended matter; Fig. 9). Considering this, it can be assumed that PCBs are transported in the aquatic environment mainly in the dissolved form, and therefore may be carried in rivers faster than other lipophilic contaminants (e.g., PAHs).

## Conclusion

Due to the presence of dissolved organic matter and colloidal and suspended fractions, surface waters constitute an interactive multiphase system, in which organic compounds occur in dissolved and adsorbed forms. The results derived from determinations of the total content of organic pollutants, e.g., PCBs, in water cannot be the only source of information on the risk posed by their presence in the environment. This information can be obtained by means of a physical speciation analysis. By defining the form in which the organic pollutants occur, we can find answers to questions concerning the potential hazard of these compounds to the environment and their possible influence on living organisms.

The results of this study allow us to conclude that due to the hydrophobic character and low volatility, PCBs display the following characteristics:

1. In low-energy environments, the dominant form of PCBs is associated with suspended and colloidal fractions, whereby the partition coefficient of the concentration of these compounds between the sediment and aqueous phase is 10^3^–10^4^ l/kg.
2. PCBs are transported in high-energy aquatic environments (streams and rivers) mainly in a dissolved form.
3. Desorption and transition of PCBs from the solid matter into the aqueous phase appears to be limited and the levels of dissolved target analytes are negligibly small (<0.5 %) compared to their solubility in water. This may indicate that the risk of secondary water pollution associated with remobilization of PCBs is low.

The results of the present study may be of great importance for monitoring PCB concentrations in running waters. They may help optimize the monitoring procedures for aquatic ecosystems (location, frequency, and matrix), and may subsequently reduce costs of analysis. Studies on remobilization of PCBs may also be of great significance for planning and management of anthropogenic activities, such as dredging, dredge disposal, and fishing in the most vulnerable ecosystems.
